# Mobile Breast Cancer e-Support Program for Chinese Women With Breast Cancer Undergoing Chemotherapy (Part 3): Secondary Data Analysis

**DOI:** 10.2196/18896

**Published:** 2020-09-16

**Authors:** Haihua Zhu, Xiuwan Chen, Jinqiu Yang, Qiaoling Wu, Jiemin Zhu, Sally Wai-Chi Chan

**Affiliations:** 1 Nursing Department First Affiliated Hospital Xiamen University Xiamen China; 2 Department of Nursing, School of Medicine Xiamen University Xiamen China; 3 Hospital Infection Management Office China-Japan Friendship Hospital Beijing China; 4 Global Engagement and Partnership Division, UON Singapore campus University of Newcastle Newcastle Australia

**Keywords:** breast cancer, chemotherapy, mobile app, mHealth

## Abstract

**Background:**

Many app-based interventions targeting women with breast cancer have been developed and tested for effectiveness. However, information regarding the evaluation of the usage of these interventions is scarce. A better understanding of usage data is important to determine how women use apps and how these interventions affect health outcomes.

**Objective:**

This study aimed to examine the usage duration and login frequency of an app-based intervention, the Breast Cancer e-Support (BCS) program, and to investigate the association between usage data and participants’ demographic and medical characteristics.

**Methods:**

This study is a secondary data analysis of a randomized controlled trial assessing the effectiveness of the BCS program. The BCS program contains four modules: Learning Forum, Discussion Forum, Ask-the-Expert Forum, and Your Story Forum. A total of 57 women in the intervention group accessed the BCS program during their 12-week chemotherapy. The app’s background system tracked the usage duration and login frequency for each forum and the entire BCS program.

**Results:**

The total usage duration per participant ranged from 0 to 9371 minutes, and the login frequency per participant ranged from 0 to 774 times. The Discussion Forum and the Learning Forum were the most frequently used modules. The general linear model showed that age, education, family monthly income, and employment were associated with BCS usage duration and/or login frequency. Age (*F*_1,45_=10.09, *P*=.003, *B*=115.34, 95% CI 42.22-188.47) and education level (*F*_1,45_=7.22, *P*=.01, *B*=1949.63, 95% CI 487.76-3411.50) were positively associated with the usage duration of the entire BCS program. Family monthly income was positively associated with the usage duration of the Learning Forum (*F*_1,45_=11.85, *P*=.001, *B*=1488.55, 95% CI 617.58-2359.51) and the login frequency of the entire BCS program (*F*_1,45_=4.47, *P*=.04, *B*=113.68, 95% CI 5.33-222.03). Employment was negatively associated with the usage duration of the Ask-the-expert Forum (*F*_1,45_=4.50, *P*=.04, *B*=–971.87, 95% CI –1894.66 to –49.07) and the Your Story Forum (*F*_1,45_=5.36, *P*=.03, *B*=–640.71, 95% CI –1198.30 to –83.11) and positively associated with the login frequency of the entire BCS program (*F*_1,45_=10.86, *P*=.002, *B*=192.88, 95% CI 75.01-310.74). No statistical differences were found between BCS usage data and cancer stage, BMI, comorbidity, types of surgery, or cycles of chemotherapy.

**Conclusions:**

Overall, this study found considerable variability in the usage of app-based interventions. When health care professionals incorporate app-based interventions into their routine care for women with breast cancer, the learning and discussion functions of apps should be strengthened to promote engagement. Additionally, characteristics of women with breast cancer, such as age, level of education, income, and employment status, should be taken in consideration to develop tailored apps that address their particular needs and therefore improve their engagement with the app.

**Trial Registration:**

Australian New Zealand Clinical Trials Registry ACTRN12616000639426; http://www.ANZCTR.org.au/ACTRN12616000639426.aspx

## Introduction

Chemotherapy often causes undesirable side effects in women with breast cancer, including fatigue, pain, sweating, swollen hands, and anxiety [1,2]. In China, access to adequate and continuous cancer care is challenging for women with breast cancer due to the increasing incidence of this type of cancer, shortage of specialized oncology health care professionals, and rising medical cost [3,4]. Innovative and easily accessible support is needed.

Connected Health, a health care delivery model to maximize health care resources, provides channels for participants to interact with clinicians and receive health-related services via new technology [5]. Mobile health (mHealth) is an example of this model of Connected Health [6]. Currently, thousands of health apps are available worldwide [7], with the most common type of cancer-related apps focussing on breast cancer [6].

We previously developed an app-based interactive program, called Breast Cancer e-Support (BCS) [8]. This program could improve women’s self-efficacy and quality of life as well as reduce symptom interference during chemotherapy through 12-week access [9]. Women also perceived the BCS program to be beneficial in enriching knowledge, promoting confidence level, and improving emotional well-being [10]. Additionally, BCS usage duration was positively related to women’s self-efficacy, social support, and quality of life during chemotherapy [9], which is consistent with a previous finding: “the more use, the better health outcome” [11].

A better understanding of app usage is critical to elucidate women’s preferences for different forums and the ways the BCS program might affect health outcomes [12]. The usage statistics from an app are the real-world representations of individual usage processes [12], which are related to women’s app perceptions and usage experience [13]. The evaluation of usage data might help to provide design recommendations to improve app engagement [13]. However, information on app usage, including duration and frequency, remains scarce [14-16]. Furthermore, it is unknown what modules are the most popular among participants.

Women with different characteristics, such as demographic and health related factors, are expected to have different usage patterns for app-based interventions [17]. Therefore, tailored interventions could be more effective and acceptable for women with breast cancer [18,19]. However, successful tailoring is challenging because of the heterogeneous characteristics of women with breast cancer [17,18,20,21]. Currently, findings are still inconclusive on the relationship between women’s characteristics and their preference on app use [17,21-25]. For example, a recent systematic review revealed contrasting findings on whether age and baseline symptom status are associated with app usage [20]. A better understanding of the associations between women’s characteristics and their usage patterns of the BCS program is critical to improve the design of app-based interventions.

The primary aim of this study was to examine the usage data of the BCS program by assessing the usage duration and login frequency in women randomized in an intervention group. The secondary aim was to investigate the associations between usage data and demographic and medical characteristics.

## Methods

### Participants

This study extracted data from a multicenter randomized control trial and focused on the analysis of 57 women randomly assigned to the BCS intervention group. All participants had to (1) be diagnosed with breast cancer, (2) be receiving chemotherapy, and (3) have access to the BCS program for 12 weeks. Women who had a concurrent serious physical illness or chronic mental illness were excluded from the study.

### Intervention

The BCS app program, which could be used on both iPhone and Android mobile phones, aimed to provide information and social support to improve women's symptom management during chemotherapy. The intervention was conducted from May to November 2016. The full detailed description of the BCS program has been published [26].

The BCS program has four modules: Learning Forum, Discussion Forum, Ask-the-Expert Forum, and Your Story Forum. The Learning Forum offers evidence-based knowledge of breast cancer and management strategies for related symptoms. The Discussion Forum provides an anonymous interaction platform for women to communicate with peers and health care professionals. A seasoned health care professional moderates online discussion and provides expert advice when needed. The Ask-the-Expert Forum provides health consultation, where health care professionals respond to women’s queries within 24 hours. The Your Story Forum presents videos of encouraging stories that include strategies for overcoming the challenges during chemotherapy [26]. A moderator facilitates the interaction between peers and health care professionals in the BCS program.

### Demographic and Medical Characteristics

At baseline, women filled out paper questionnaires that collected demographic characteristics including age; marital status; education; employment; family income; BMI; and medical characteristics such as cancer stage, types of surgery, comorbidity, complications, and cycles of chemotherapy. Medical records were used to confirm the medical variables.

### Usage Data: Usage Duration and Login Frequency

The usage data of the BCS program were measured as usage duration and login frequency; data from each forum and the entire BCS program were recorded for each individual for 12 weeks. Usage duration was defined as the time, in minutes, between login and logout for each forum and the entire BCS program. The operational definition of login frequency was the number of times that each woman logged into each forum and the entire BCS program during the 12-week intervention period. The app’s background system tracked the usage data. If women were surfing on other functionalities of mobile phones or mobile phones were in standby mode, the app ran on background operational mode and the app background thread stopped calculating the usage data.

### Statistical Analyses

All data analyses were performed using IBM SPSS 25.0. The mean (SD), medium (IQR), and maximum values were used to describe usage duration and login frequency. As the usage data were highly skewed, a general linear model was used to calculate the associations between usage data and demographic and medical characteristics. An α level of .05 of statistical significance was set for all analyses.

## Results

### Demographic and Medical Characteristics

A total of 57 women were included in this study. The mean age was 46.2 years (SD 8.5 years). All women were married, and 77% (44/57) were unemployed at the time of the study. Regarding educational level, 28% (16/57) had completed middle school, followed by elementary school (13/57, 23%), and high school (12/57, 21%). The monthly family income was in the range of US $149 to $738 for 60% (34/57) of the women and <US $148 for 25% (14/57) of them. Many of the women (28/57, 49%) were diagnosed with stage II breast cancer, followed by stage III breast cancer (19/57, 33%). A majority (45/57, 79%) had undergone mastectomy and only a few (3/57, 5%) had undergone breast conserving surgery.

### BCS Usage Duration and Login Frequency

BCS usage data showed great variability. Only 4% (2/57) of the women did not log in the BCS program. For the whole BCS program, usage duration ranged from 0 to 9371 minutes, and frequency of logins varied from 0 to 774 times. The Discussion Forum and the Learning Forum were the most popular forums for women to log in and use. Table 1 shows the large difference between the mean and median of the BCS usage statistics.

**Table 1:** Usage duration and login frequency of Breast Cancer e-Support program for women with breast cancer receiving chemotherapy during the 12-week intervention (n=57).

Program and forums	Usage duration (minutes)			Login frequency (times)			
	Mean (SD)	Medium (IQR)	Maximum		Mean (SD)	Medium (IQR)	Maximum
Entire BCS^a^ Program	1072.33 (2359.48)	100 (27-279)	9371		54.7 (131.4)	11 (5-27)	774
Learning Forum	399.51 (1139.95)	29 (12-162)	5926		13.75 (20.54)	6 (3-15)	95
Discussion Forum	412.09 (1364.85)	4 (1-43)	8539		53.89 (130.9)	7 (3-28)	715
Ask-the-Expert Forum	161.91 (1013.8)	2 (0-8)	7652		13.05 (25.26)	4 (2-12)	132
Your Story Forum	98.84 (597.73)	1 (0-13)	4480		5.18 (8.06)	3 (1-7)	52

^a^ BCS: Breast Cancer Support.

### Associations Between BCS Usage Data and Demographic and Medical Characteristics

Age, education, family monthly income, and employment were associated with BCS usage duration and/or login frequency. Age was positively associated with the usage duration of the entire BCS program (*F*_1,45_=10.09, *P*=.003, *B*=115.34, 95% CI 42.22-188.47) and the Learning Forum (*F*_1,45_=7.71, *P*=.008, *B*=49.93, 95% CI 13.70-86.13) (Table 2). Education level was found to be positively associated with the usage duration of the entire BCS program (*F*_1,45_=7.22, *P*=.01, *B*=1949.63, 95% CI 487.76-3411.50) and the Discussion Forum (*F*_1,45_=7.45, *P*=.01, *B* =1303.24, 95% CI 341.27-2265.21) (Table 2) as well as the login frequency of the Learning Forum (*F*_1,45_=7.07, *P*=.01, *B*=17.82, 95% CI 4.32-31.32) and the Ask-the-expert Forum (*F*_1,45_=6.17, *P*=.02, *B*=21.01, 95% CI 3.97-38.06) (Table 3). Family monthly income was positively associated with the usage duration of the Learning Forum (*F*_1,45_=11.85, *P*=.001, *B*=1488.55, 95% CI 617.58-2359.51) (Table 2) and the login frequency of the entire BCS program (*F*_1,45_=4.47, *P*=.04, *B*=113.68, 95% CI 5.33-222.03), the Learning Forum (*F*_1,45_=4.61, *P*=.04, *B*=17.31, 95% CI 1.07-33.56), and the Discussion Forum (*F*_1,45_=6.68, *P*=.01, *B*=137.06, 95% CI 30.21-243.91) (Table 3). Employment status was negatively associated with the usage duration of the Ask-the-expert Forum (*F*_1,45_=4.50, *P*=.04, *B*=–971.87, 95% CI –1894.66 to –49.07) and the Your Story Forum (*F*_1,45_=5.36, *P*=.03, *B*=–640.71, 95% CI –1198.30 to –83.11) (Table 2) and positively associated with the login frequency of the entire BCS program (*F*_1,45_=10.86, *P*=.002, *B*=192.88, 95% CI 75.01-310.74), the Learning Forum (*F*_1,45_=11.76, *P*=.001, *B*=30.08, 95% CI 12.41-47.75), the Discussion Forum (*F*_1,45_=10.31, *P*=.002, *B*=185.32, 95% CI 69.08-301.55), and the Your Story Forum (*F*_1,45_=6.76, *P*=.01, *B*=9.30, 95% CI 2.09-16.51) (Table 3). No statistical differences were found between BCS usage data and cancer stage, BMI, comorbidity, types of surgery, or cycles of chemotherapy.

**Table 2:** Associations between usage duration in Breast Cancer e-Support program and demographic and clinical characteristics (n=57).

Variables		Usage duration																			
		Entire BCS program				Learning Forum				Discussion Forum				Ask-the-Expert Forum				Your Story Forum			
		*F* test (*df*)		*P* value		*F* test (*df*)		*P* value		*F* test (*df*)		*P* value		*F* test (*df*)		*P* value		*F* test (*df*)		*P* value	
Age		10.09 (1,45)		.003^d^		7.71 (1,45)		.008^d^		2.46 (1,45)		.12		.70 (1,45)		.41		1.60 (1,45)		.21	
Education^a^		7.22 (1,45)		.01^d^		2.17 (1,45)		.15		7.45 (1,45)		.01^d^		.14 (1,45)		.72		.003 (1,45)		.96	
Family monthly income^b^		3.50 (1,45)		.07		11.85 (1,45)		.001^d^		.001 (1,45)		.98		.38 (1,45)		.54		2.68 (1,45)		.11	
Cancer stage^c^		1.06 (1,45)		.31		.72 (1,45)		.40		3.30 (1,45)		.08		2.73 (1,45)		.11		.52 (1,45)		.47	
	BMI		.29 (1,45)		.60		.28 (1,45)		.60		.21 (1,45)		.65		.30 (1,45)		.59		1.08 (1,45)		.31
Employment		.04 (1,45)		.85		2.91 (1,45)		.10		.99 (1,45)		.32		4.50 (1,45)		.04^d^		5.36 (1,45)		.03^d^	
Comorbidity		.44 (1,45)		.51		.001 (1,45)		.97		.12 (1,45)		.73		1.21 (1,45)		.28		1.72 (1,45)		.20	
Types of surgery		.27 (2,45)		.76		.72 (2,45)		.49		.59 (2,45)		.56		.99 (2,45)		.38		.81 (2,45)		.45	
Cycles of chemotherapy		.81 (2,45)		.45		2.76 (2,45)		.07		.56 (2,45)		.57		1.86 (2,45)		.17		.85 (2,45)		.44	

^a^Regrouped into two categories: education “0=junior middle school and lower”, “1=higher school and higher.”

^b^Regrouped into two categories: family monthly income “0<USD 442”, “1≥USD 442.”

^c^Regrouped into two categories: “0=cancer stage I and II”, “1=cancer stage III or IV.”

^d^Significant at an α level of .05.

**Table 3:** Associations between login frequency in Breast Cancer eSupport program and demographic and clinical characteristics (n=57).

Variables	Login frequency													
	Login times: Entire BCS program			Login times: Learning Forum			Login times: Discussion Forum			Login times: Ask-the-Expert Forum			Login times: Your Story Forum	
	*F* test (*df*)	*P* value	*F* test (*df*)		*P* value	*F* test (*df*)		*P* value	*F* test (*df*)		*P* value	*F* test (*df*)		*P* value
Age	.01 (1,45)	.91	1.39 (1,45)		.25	.51 (1,45)		.48	.36 (1,45)		.55	1.04 (1,45)		.31
Education^a^	3.61 (1,45)	.06	7.07 (1,45)		.01^d^	2.54 (1,45)		.12	6.17 (1,45)		.02^d^	2.19 (1,45)		.15
Family monthly income^b^	4.47 (1,45)	.04^d^	4.61 (1,45)		.04^d^	6.68 (1,45)		.01^d^	.04 (1,45)		.84	3.75 (1,45)		.06
Cancer stage^c^	.61 (1,45)	.44	1.71 (1,45)		.20	1.13 (1,45)		.29	1.69 (1,45)		.20	1.41 (1,45)		.24
Body mass index	.72 (1,45)	.40	.29 (1,45)		.59	.79 (1,45)		.38	2.11 (1,45)		.15	2.89 (1,45)		.10
Employment	10.86 (1,45)	.002^d^	11.76 (1,45)		.001^d^	10.31 (1,45)		.002^d^	3.66 (1,45)		.06	6.76 (1,45)		.01^d^
Comorbidity	1.48 (1,45)	.23	.95 (1,45)		.34	1.10 (1,45)		.30	.02 (1,45)		.90	.35 (1,45)		.57
Types of surgery	1.80 (2,45)	.18	1.48 (2,45)		.24	1.05 (2,45)		.36	.64 (2,45)		.53	1.58 (2,45)		.22
Cycles of chemotherapy	.50 (2,45)	.61	.43 (2,45)		.66	.35 (2,45)		.70	3.25 (2,45)		.05	.54 (2,45)		.59

^a^Regrouped into two categories: education “0=junior middle school and lower”, “1=higher school and higher.”

^b^Regrouped into two categories: family monthly income “0<USD 442”, “1≥USD 442.”

^c^Regrouped into two categories: “0=cancer stage I and II”, “1=cancer stage III or IV.”

^d^Significant at an α level of .05.

## Discussion

### Principal Findings

In this study, we found considerable variability in usage duration and frequency of the BCS program among women with breast cancer receiving chemotherapy. The large difference between median and mean usage data indicated usage polarization among the women. Additionally, we found that the Discussion Forum and the Learning Forum were the most popular forums. Age, education, family income, and employment were associated with women’s usage data.

Although BCS usage varied considerably, this study reported better usage duration and login frequency than a self-guided 4-month web-based intervention, called BREAst cancer ehealTH (BREATH), in which the total usage duration ranged from 0 to 2324 minutes (mean 337.2 minutes, SD 163.7 minutes) and the frequency of login ranged from 0 to 45 times (mean 11 times, SD 47 times) [27]. Moreover, our study had a lower proportion of non-users (2/57, 4%) than the BREATH intervention (7/70, 10%) [27]. BREATH, as a self-guided intervention, did not have professionals to moderate it. In contrast, this study involved a health care moderator who facilitated group interactions in the Discussion Forum and sent reminder messages to the corresponding doctors in the Ask-the-Expert Forum [9]. To explore whether a moderator is helpful in increasing participants’ engagement, further studies are warranted to compare usage statistics across different formats (self-guided versus moderated) within the same program.

We found usage polarization in the BCS program, which is consistent with the results of a study that explored the usage data of a web-based self-management intervention [27]. Usage polarization may reflect the particular preferences of each participant. In cases where reduced app engagement may undermine the potential effectiveness of the interventions [9], engaging strategies should be developed, such as self-tailored content, targeting participants who do not use the app as often as expected. However, tailoring the intervention to meet each individual’s needs presents multiple challenges [27]. Additionally, our process evaluation of the BCS program indicated that women’s poor physical and psychological conditions might hamper BCS usage [10]. Furthermore, in China, family members are often considered the primary caregivers of patients [28]. Therefore, to achieve better health outcomes for patients, family members should be involved in app-based interventions and information should be supplemented for them.

In this study, women used the Learning Forum and the Discussion Forum the most during their 12-week access to BCS. Research has found that patients with cancer use mHealth as an information resource and for emotional support [11,29,30]. The Learning Forum offered evidence-based information and strategies for managing symptoms, which helped women to better cope with breast cancer [26]. Thus, information support services may contribute to the usage of mHealth [30,31]. Meanwhile, the Discussion Forum provided a multi-interaction channel for in-depth discussion with peers and health professional moderators, which held particular appeal to these women [11,18,26,32,33]. Our findings concurred with the results of other studies that women tend not to use all the modules in apps, and instead prefer those that engage them [34]. Therefore, health care professionals should strengthen the key features of apps, such as learning and discussion functions, to enhance women’s engagement.

Contrary to a previous study regarding eHealth use and age [35], we found that older women spent more time on the BCS program and the Learning Forum than younger women. In the abovementioned study, although older people were less likely to use eHealth intervention, the age disparity was found to be related to technological proficiency [35]. In this study, the user-centered design of the BCS program may help older women to alleviate their concern regarding technological ability [27]. Moreover, the interesting and relevant information gained from the Learning Forum may encourage older women to use eHealth more often [17]. Therefore, it would be beneficial to design user friendly apps and provide appropriate technical support and easy-to-understand information for older participants to meet their health information and care needs.

Consistent with some studies [20,21,23,34], our findings suggested that women with higher levels of education use the BCS program more frequently and spend more time on it. This may be interpreted as women with a higher education accepting new things more quickly and having better comprehension of the written information. These women also tend to spend more time learning health-related information [36]. Meanwhile, women with low education levels might experience difficulties in understanding the information, resulting in less interest in participating in app-based interventions [37]. However, owing to a lack of prior knowledge and health resources, women with low education levels may need to use the interventions to gain health-related information and consultation [17]. Health care professionals may improve the app design with lay language, more illustrations, and short videos to cater to the health information needs of women with different backgrounds to overcome the education barrier [10].

In this study, women with a higher family income used the Learning Forum and the Discussion Forum more than those with a lower family income, which is in agreement with other studies [17,23,37]. On the one hand, women with a low-income have less opportunities to access and use mobile devices at home and may experience stress when using the app-based program [23]. On the other hand, women with a higher family income might be able to afford spending more time to read and chat in the app-based program [17]. Besides, women with different family incomes may have different online surfing purposes. Women with a higher family income might go online to seek information, whereas women with a lower family income might seek entertainment [37]. However, low-income women with breast cancer have been reported to benefit more from web-based support because this support helps to fill the service gaps in the treatment of diseases and caters to their individual needs and preferences [38]. Thus, it is important for health care professionals to explore the needs of women with different income and improve the design and content of apps accordingly.

A previous study found that employed participants showed more interest in eHealth [35] and benefited more from eHealth [39]. Similarly, employed women used our BCS program more frequently. However, they spent less time in the Ask-the-Expert Forum and the Your-Story Forum, probably because participating in eHealth is time consuming and employed individuals have less leisure time [17]. In our process evaluation of the BCS program, women also suggested timely feedback from the experts as well as short and concise videos to convey information in these two forums [28]. These results indicate that modifying the design of eHealth to be timesaving may help facilitate usage engagement.

### Strengths and Limitations

One strength of this study was the presentation of a method that directly and objectively captured the usage data of mobile apps. Thus, there was no reporting bias for mHealth intervention usage. Another strength was that this study analyzed the usage of each module in the program and its associations with women’s demographic and medical characteristics. The findings could help researchers understand participants’ preferences and explore strategies to improve app design.

As for the limitations, the study extracted usage data from a randomized control trial, and the sample size was small, which limited the generalizability of our results. Additionally, a previous study showed that the usage of apps changes over time [32]. Given the design of BCS, we could not track the dynamic usage data during the 12-week intervention. Longitudinal research with a larger sample size is warranted to gain deeper insight into the use of app-based interventions.

### Conclusions

The insights gained from this study allow us to provide recommendations for further advances in the design and content of app-based health interventions. Overall, this study illustrated considerable variability in the usage of app-based interventions. When health care professionals incorporate app-based interventions into their routine care for women with breast cancer, the learning and discussion functions of apps should be strengthened to promote engagement. Additionally, characteristics of women with breast cancer, such as age, education level, income and employment status should be taken in consideration to develop tailored apps that address their particular needs and therefore improve their engagement with the app.

## Abbreviations

BCS: Breast Cancer e-Support
mHealth: mobile health
